# Kallikrein-Related Peptidase 4 Inhibits Laryngeal Squamous Cell Carcinoma Potentially via Involvement of the Wnt/β-Catenin Signaling Pathway

**DOI:** 10.1007/s10528-026-11443-w

**Published:** 2026-08-20

**Authors:** Jinxin Wang, Lixue Jiang, Jiahui Han, Chunguang Dong

**Affiliations:** 1 Department of Otolaryngology-Head and Neck Surgery, The First People’s Hospital of Lianyungang, No. 182, Tongguan North Road, Haizhou District, Lianyungang, Jiangsu China; 2 The Affiliated Lianyungang Hospital of Xuzhou Medical University, The First People’s Hospital of Lianyungang, Lianyungang, Jiangsu China; 3 The First Affiliated Hospital of Kangda College of Nanjing Medical University, The First People’s Hospital of Lianyungang, Lianyungang, Jiangsu China; 4 Department of Otolaryngology-Head and Neck Surgery, Lianyungang Municipal Oriental Hospital, Lianyungang, Jiangsu China

**Keywords:** Kallikrein-related peptidase 4 (KLK4), Laryngeal squamous cell carcinoma, Wnt/β-catenin signaling, Proliferation, Epithelial-mesenchymal transition

## Abstract

Laryngeal squamous cell carcinoma (LSCC) is one of the most common malignant tumors of the head and neck with an increasing incidence worldwide. As a secreted serine protease, Kallikrein-related peptidase 4 (KLK4) has been reported to exert divergent tumor-modulating roles across multiple malignancies: it acts as a tumor suppressor in some cancers while promoting progression in others. However, its role in LSCC remains unclear. The aberrant activation of Wnt/β-catenin signaling is closely linked to tumor progression in most malignancies, hence we focused on this pathway to explore the downstream mechanism of KLK4. The expression of KLK4 was analyzed in clinical samples and LSCC cell lines using reverse transcription-quantitative polymerase chain reaction (RT-qPCR) and western blotting. AMC-HN-8 cells were transfected with KLK4 plasmids, and the effects on proliferation, apoptosis, and epithelial-mesenchymal transition (EMT) were assessed using EDU assays, flow cytometry, and western blotting. The regulatory role of KLK4 on the Wnt/β-catenin signaling pathway was further investigated by treating cells with the Wnt/β-catenin agonist SKL2001. KLK4 expression was significantly downregulated in LSCC clinical samples and cell lines. KLK4 overexpression in LSCC cells inhibited cell proliferation, promoted apoptosis, and reversed the abnormal expression of EMT-related molecular markers. Moreover, KLK4 overexpression modulated the Wnt/β-catenin signaling by downregulating Wnt3a, β-catenin, and downstream targets. Treatment with SKL2001 reversed these effects by restoring cell proliferation, reducing apoptosis, and promoting EMT. KLK4 modulates LSCC cell proliferation, apoptosis and the expression of EMT-related markers, which are associated with the activity of the Wnt/β-catenin signaling pathway.

**Supplementary Information:**

The online version contains supplementary material available at https://doi.org/10.1007/s10528-026-11443-w.

## Introduction

Head and neck squamous cell carcinoma (HNSCC) is a type of cancer with increasing incidence worldwide, and LSCC is one of its primary subtypes (Johnson et al. 2020). LSCC is characterized by extensive genomic instability, including chromosomal instability, polyploidy/aneuploidy, and specific genetic abnormalities, such as oncogene amplification, point mutations, and tumor suppressor gene deletions, as well as epigenetic changes, such as promoter methylation and miRNA dysregulation (Mastronikolis et al. 2020). The main risk factors for LSCC include smoking, alcohol consumption, betel nut use, and high-risk human papillomavirus (hr HPV) (Tsiambas et al. 2020; Matta and Ralhan 2009). Although various treatment options are available, the overall survival rate of LSCC has declined in recent decades because most patients are diagnosed at an advanced stage (Wang et al. 2021). Therefore, investigating the molecular mechanisms underlying LSCC and identifying reliable diagnostic and therapeutic biomarkers are crucial.

Research indicates that the development and progression of LSCC are closely related to the dysregulation of multiple molecular signaling pathways, among which the abnormal activation of the Wnt/β-catenin signaling pathway is widely recognized as a key driver of tumorigenesis (Li et al. 2020; Xie et al. 2021; Shiah et al. 2015). The Wnt/β-catenin signaling pathway plays a crucial role in normal cellular development and tissue homeostasis, maintaining proper cell proliferation and differentiation (Ma et al. 2023). However, the aberrant activation of this pathway often leads to cancer initiation and malignant progression (Zhang and Wang 2020). Research has demonstrated that the Wnt/β-catenin signaling pathway is aberrantly activated in LSCC and that this dysregulation is closely linked to poor patient prognosis (Sun et al. 2018). Meng et al. reported that chromobox protein homolog 8 (CBX8) promotes LSCC cell proliferation and migration while inhibiting apoptosis by activating the Wnt/β-catenin pathway and inducing the EMT process, thereby contributing to adverse clinical outcomes (Meng et al. 2022). Identifying upstream effector proteases that modulate Wnt/β-catenin cascade may uncover novel regulatory mechanisms underlying LSCC progression.

KLK4 is a member of the serine protease family (Kontos and Scorilas 2012). Proteases of the KLK family play crucial roles in various physiological and pathological processes, including blood pressure regulation, skin desquamation, and cancer development and progression (Avgeris et al. 2010; Borgoño and Diamandis 2004). KLK4 displays highly context-dependent functions across malignancies: its upregulation drives invasiveness and unfavorable prognosis in prostate and ovarian carcinomas (Wang et al. 2018; Klokk et al. 2007), while one clinical cohort analysis has documented markedly reduced KLK4 mRNA in primary LSCC lesions relative to paired peritumoral tissues (Foteinou et al. 2014). Functionally, KLK4 modulates core malignant phenotypes including proliferation, apoptosis and EMT via crosstalk with multiple signaling cascades in different tumor backgrounds (Yang et al. 2014).

The Wnt/β-catenin signaling pathway is a crucial regulator of cell proliferation, differentiation, and apoptosis, and plays a key role in the development and progression of various cancers (Ma et al. 2023; Zhang and Wang 2020; He and Tang 2020). Despite the documented expression difference of KLK4 in LSCC tissue, the biological activity of KLK4 and its potential regulatory connection to the Wnt/β-catenin pathway have never undergone systematic characterization in laryngeal cancer.

Given the aberrant expression of KLK4 observed across multiple solid tumors, numerous studies have assessed its utility as a diagnostic or prognostic biomarker tracking malignant progression. For instance, differential KLK4 expression correlates with tumor stage, metastatic burden and overall patient survival in prostate, ovarian, and oral squamous cell carcinomas, confirming its biomarker relevance across these malignancies. Despite these findings in other tumor types, whether KLK4 expression can serve as a candidate biomarker to reflect LSCC disease progression remains uninvestigated to date. This unaddressed clinical question further motivates our present exploration of KLK4 function in laryngeal cancer.

The aim of this study was to investigate the specific role of KLK4 in LSCC, particularly its regulatory effect on the Wnt/β-catenin signaling pathway. By elucidating the interaction between KLK4 and the Wnt/β-catenin pathway and its impact on tumor progression, this research will contribute to a deeper understanding of the molecular pathogenesis of LSCC. These findings provide preliminary experimental clues for subsequent large-cohort validation of KLK4 as a candidate biomarker and prospective therapeutic target for LSCC.

## Materials and Methods

### Collection and Processing of Clinical Samples

Serum samples and cancerous and adjacent normal tissue samples were collected from 30 patients with LSCC. Serum samples from 30 healthy volunteers served as the controls. All tissue samples were snap-frozen in liquid nitrogen and stored at − 80 °C for future analysis. Serum samples were centrifuged and stored at − 80 °C for subsequent gene and protein expression analyses. All procedures involving human participants were performed in accordance with the ethical standards of the institutional and/or national research councils, and the 1964 Declaration of Helsinki and its subsequent amendments or similar ethical standards. The study design was approved by the Ethics Committee of Lianyungang First People’s Hospital (Approval number: LW-20251027003-01). Informed consent was obtained from all the participants.

### Cell Culture and Transfection

Three LSCC cell lines (AMC-HN-8, TU177, and TU686) and a normal nasopharyngeal epithelial cell line, NP69, were purchased from the American Type Culture Collection (ATCC). Cells were cultured in Dulbecco’s modified Eagle’s medium (DMEM) supplemented with 10% fetal bovine serum (FBS) at 37 °C in a 5% CO₂ incubator.

1 µg KLK4-plasmid (sc-404256-ACT Santa Cruz Biotechnology) and 1 µg control plasmids (sc-437275 Santa Cruz Biotechnology) were transfected into AMC-HN-8 cells (2 × 10⁵ cells per well 24 well plates) usingLipofectamine 3000 (Thermo Fisher, L3000015) according to the manufacturer’s instructions. After 24 h of transfection, the Wnt/β-catenin pathway agonist SKL2001 (40 µM) was added to the transfected cells and incubated for another 24 h to investigate the regulatory effects of KLK4 on this pathway.

### RNA Extraction and Reverse Transcription-Quantitative Polymerase Chain Reaction (RT-qPCR) Analysis

Total RNA was extracted from the cells (1 × 10⁶ cells) and tissue samples using TRIzol reagent (Yifei Xue Biotechnology, YFXM0011P) according to the manufacturer’s instructions. cDNA was synthesized using HiScript III RT SuperMix for qPCR (+ gDNA wiper) (Vazyme, R323). RT-qPCR was performed using the ChamQ Universal SYBR qPCR Master Mix kit (Q711-02; Vazyme) to detect the mRNA expression levels of KLK4, E-cadherin, and N-cadherin. Each sample was tested in triplicate, and GAPDH was used as an internal control. The relative expression levels were calculated using the 2^−ΔΔCt^ method. Primer sequences are listed in Table 1. The amplification efficiency of each primer pair was calculated and verified (KLK4: 98.2%; E-cadherin: 96.7%; N-cadherin: 99.1%; GAPDH: 97.5%).

**Table 1:** The RT-qPCR primer sequences

Name		Sequence (5′–3′)
Homo GAPDH	Sense	AGGTCGGAGTCAACGGATTT
	Antisense	TGACGGTGCCATGGAATTTG
Homo KLK4	Sense	AGCTCTATGACCCGCTGTAC
	Antisense	TGCAGAGGTTGGTGTAGACA
Homo E-cadherin	Sense	ACGCATTGCCACATACACTC
	Antisense	GGTGTTCACATCATCGTCCG
Homo N-cadherin	Sense	CGGTTTCATTTGAGGGCACA
	Antisense	TTGGAGCCTGAGACACGATT

### Western Blot Analysis

Total protein was extracted from cells (2 × 10⁶ cells/well, 6 well plates) and tissue samples using RIPA lysis buffer, and protein concentrations were measured using the BCA assay. Equal amounts of protein (30 µg of total protein) were separated by sodium dodecyl sulfate-polyacrylamide gel electrophoresis and transferred to PVDF membranes. The membranes were incubated with primary antibodies specific to KLK4 (ab181402, dilution 1:1000, Abcam), Wnt3a (26744-1-AP, dilution 1:1000, Proteintech), β-catenin (51067-2-AP, dilution 1:5000, Proteintech), p-GSK3β (67558-1-Ig, dilution 1:2000, Proteintech), GSK3β (22104-1-AP, dilution 1:2000, Proteintech), C-Myc (18583, dilution 1:1000, CST), Cyclin D1 (60186-1-Ig, dilution 1:5000, Proteintech), cleaved-Caspase3 (25128-1-AP, dilution 1:1000, Proteintech), E-cadherin (20874-1-AP, dilution 1:20000, Proteintech), N-cadherin (22018-1-AP, dilution 1:2000, Proteintech), and GAPDH (60004-1-Ig, 1:50000, Proteintech), followed by HRP-conjugated secondary antibodies (7076/7074, 1:1000, CST). The protein bands were visualized using enhanced chemiluminescence (ECL) detection. The protein blot gray values were quantified using ImageJ V1.53c, with band intensity normalization to GAPDH.

### Cell Proliferation Assay

Cell proliferation was detected using the BeyoClick EdU-488 cell proliferation assay kit (Beyotime, C0071S) according to the manufacturer’s instructions. Briefly, AMC-HN-8 cells (2 × 10⁵ cells per well) were seeded in a 6-well plate overnight before the experiment and treated with drugs or stimuli as required. Subsequently, EdU working solution with 2x concentration (a final concentration of 10 µM) was added and incubated at 37 ℃ for 2 h to label proliferating cells. After incubation, the cells were fixed with a fixative for 15 min, washed three times, treated with permeabilization buffer (0.3% Triton X-100) for 15 min, and washed again. The Click-iT reaction solution was added to each well, incubated for 30 min, and washed three times after the reaction. Nuclei were stained with Hoechst 33,342. The proportion of EdU-positive cells among total Hoechst-labelled nuclei was calculated as the proliferation rate.

### Cell Apoptosis Analysis with Flow Cytometry (FCM)

Apoptosis was measured using the Annexin V-FITC/PI Apoptosis Detection Kit (Beyotime, C1062M). Cells (1 × 10⁵ cells) from the different treatment groups were collected, washed twice with PBS, and resuspended in 1× binding buffer. Annexin V-FITC and PI were added to the cells, which were then incubated in the dark at room temperature for 15 min. Apoptosis was analyzed using flow cytometry, and the proportions of early apoptotic (annexin V-positive/PI-negative) and late apoptotic (annexin V-positive/PI-positive) cells were quantified using FlowJo V10.8.1.

### Statistical Analysis

Data are presented as mean ± standard deviation (SD). Statistical comparisons were conducted using one-way ANOVA, followed by Tukey’s post hoc test for multiple group comparisons. Statistical significance was set at *p* < 0.05. All experiments were performed with three biological replicates.

## Results

### Expression of KLK4 in Clinical Samples of LSCC

**Fig. 1:**
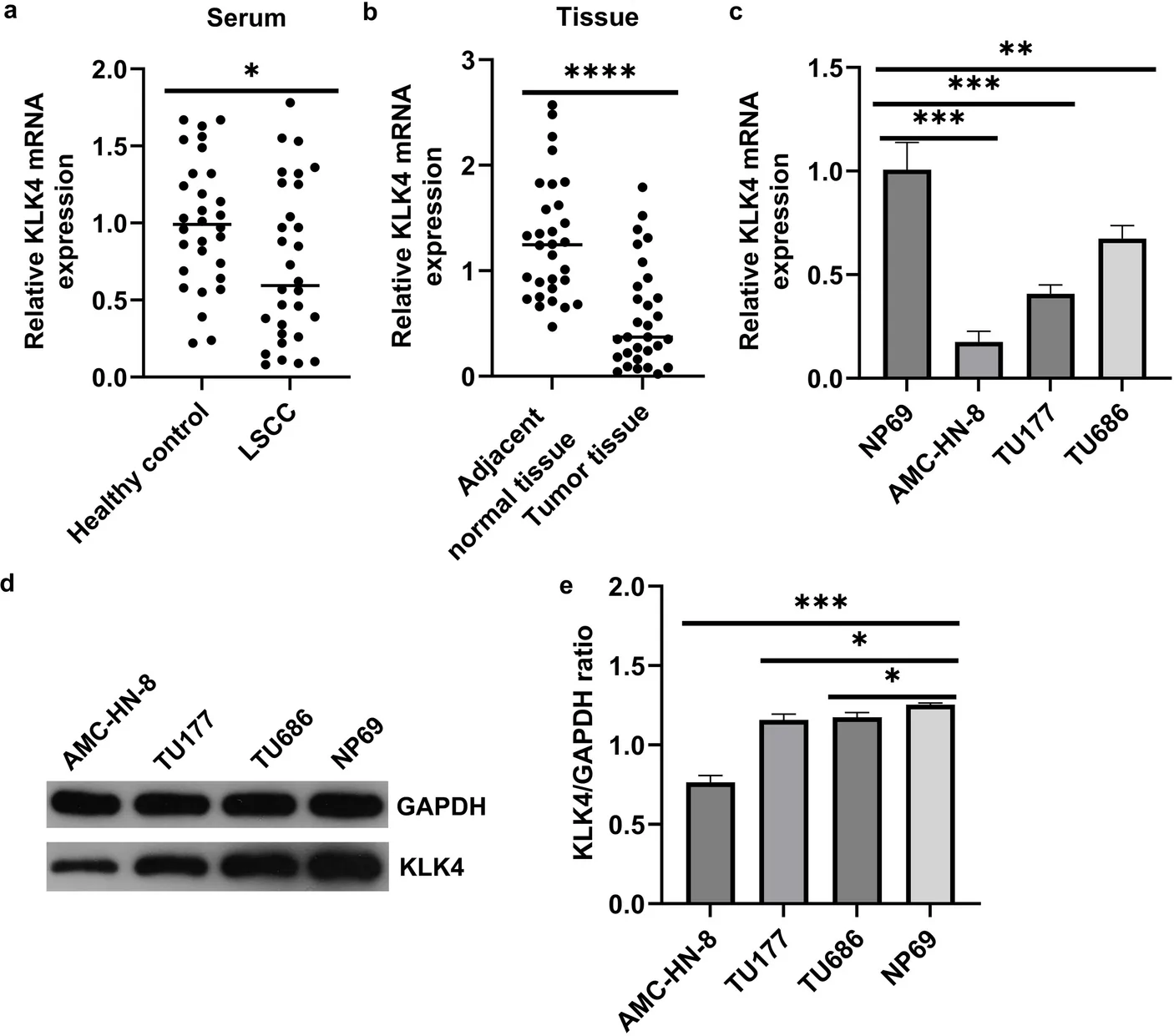
KLK4 expression in LSCC clinical samples and cell lines. **a** KLK4 mRNA levels in serum from 30 patients with LSCC and 30 healthy volunteers were quantified using RT-qPCR; **b** KLK4 mRNA expression in tumor tissues and adjacent normal tissues from 30 patients with LSCC was analyzed using RT-qPCR; **c** RT-qPCR analysis of KLK4 mRNA levels in LSCC cell lines (AMC-HN-8, TU177, TU686) and normal NP69 cells (immortalized normal human laryngeal epithelial control cells); **d** Western blot analysis of KLK4 protein levels in LSCC cell lines (AMC-HN-8, TU177, TU686) and normal NP69 cells. Data are from three independent experiments (*n* = 3). *p < 0.05; **p < 0.01; ***p < 0.001; ****p < 0.0001

This study analyzed the expression levels of KLK4 in serum samples from 30 patients with LSCC and 30 healthy volunteers, as well as in cancerous and adjacent normal tissues from 30 patients with LSCC. The RT-qPCR results showed that the relative expression of KLK4 in the serum of patients with LSCC was significantly lower, approximately one-fourth that in the healthy group (*p* < 0.05; Fig. 1a). Further comparison between cancerous and adjacent normal tissues revealed that KLK4 expression in cancerous tissues was significantly reduced, with a similar four-fold decrease compared with adjacent normal tissues (*p* < 0.0001, Fig. 1b). These findings indicate that KLK4 expression is markedly suppressed in LSCC, revealing a significant correlative relationship between reduced KLK4 abundance and LSCC pathogenesis.

### Expression of KLK4 in LSCC Cell Lines

We subsequently examined the expression of KLK4 in three LSCC cell lines (AMC-HN-8, TU177, and TU686), using the normal nasopharyngeal epithelial cell line NP69 as a control. RT-qPCR results showed that KLK4 mRNA expression was significantly downregulated in all three LSCC cell lines compared to that in control NP69 cells (Fig. 1c). Western blot analysis further confirmed the reduction in KLK4 protein levels in LSCC cell lines (Fig. 1d and e). These findings suggested that KLK4 expression was downregulated in LSCC cell lines, indicating its potential role in LSCC pathogenesis.

### The Effect of KLK4 on Proliferation, Apoptosis, and EMT in LSCC Cells

**Fig. 2:**
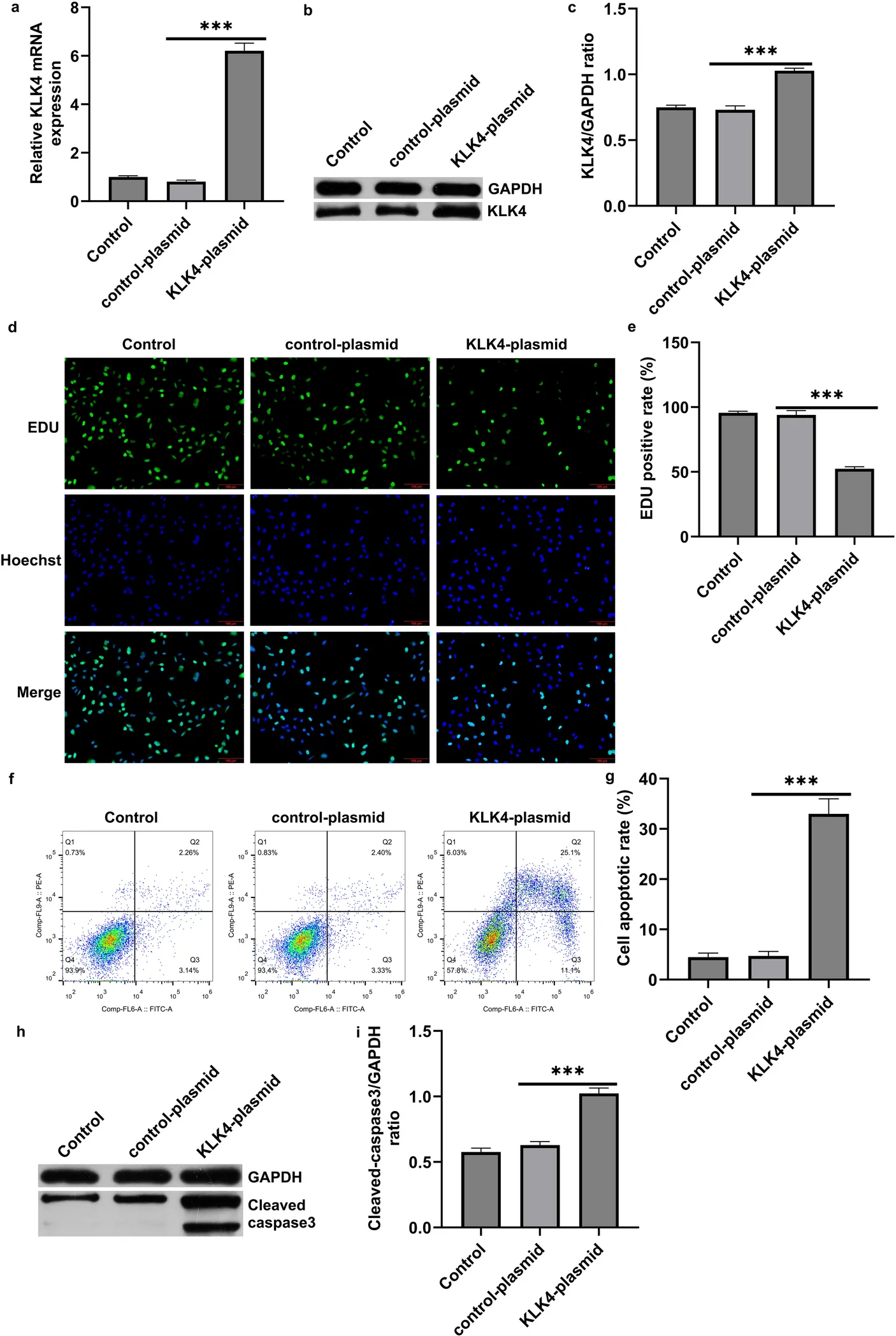
Effects of KLK4 on proliferation and apoptosis in LSCC cells. **a**–**c** RT-qPCR and western blot analysis of KLK4 mRNA and protein levels; **d** and **e** EdU assay showing the cell proliferation in AMC-HN-8 cells; **f** Flow cytometry analysis of apoptosis rates; **g** Quantification of total apoptotic cell percentage (sum of early apoptotic and late apoptotic populations) detected by Annexin V/PI flow cytometry; **h** and **i** Western blot analyses of cleaved-Caspase3 protein levels. Data are from three independent experiments (*n* = 3). ***p < 0.001

**Fig. 3:**
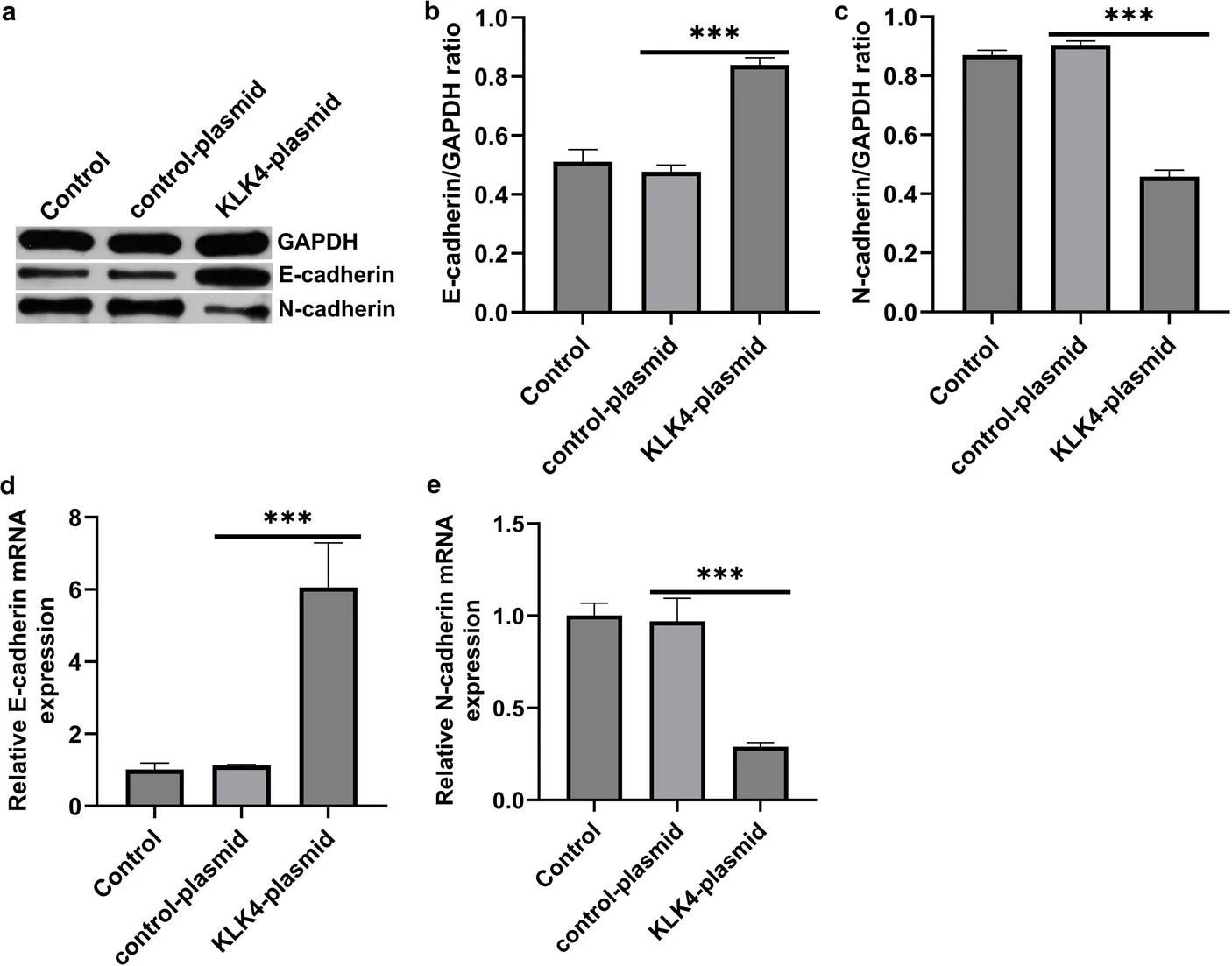
Effects of KLK4 on the expression of EMT-associated markers in LSCC cells. **a** Western blot analysis of E-cadherin and N-cadherin expression in AMC-HN-8 cells; **b** E-cadherin/GAPDH ratio in AMC-HN-8 cells; **c** N-cadherin/GAPDH ratio in AMC-HN-8 cells; **d** and **e** RT-qPCR analysis of E-cadherin and N-cadherin expression in AMC-HN-8 cells. Data are from three independent experiments (*n* = 3). ***p < 0.001

We transfected AMC-HN-8 cells with control and KLK4 plasmids, respectively, to investigate the functional impact of KLK4 on LSCC cells. First, KLK4 expression was analyzed using RT-qPCR and Western blotting. The results showed that KLK4 expression was significantly elevated by 5-fold in the KLK4 plasmid group compared to that in the control and control plasmid groups (Fig. 2a–c). Next, we assessed cell proliferation using the EdU assay. The results indicated that cell proliferation in the KLK4 plasmid group was significantly lower than that in the control plasmid group, suggesting that KLK4 overexpression inhibited LSCC cell proliferation (Fig. 2d and e). In addition, apoptosis was evaluated using flow cytometry, which showed that the apoptosis rate in the KLK4 plasmid group was significantly higher than that in the control plasmid group (Figs. 2f and g). Western blotting analysis further confirmed this by revealing a significant increase in cleaved-Caspase 3 expression in the KLK4 plasmid group, indicating that KLK4 overexpression promoted cell apoptosis (Fig. 2h and i). Finally, to assess the effect of KLK4 on EMT, we analyzed the expression levels of E-cadherin and N-cadherin using RT-qPCR and western blotting. The results demonstrated that E-cadherin, an epithelial marker, was significantly upregulated, whereas N-cadherin, a mesenchymal marker, was significantly downregulated in the KLK4 plasmid group (Fig. 3a–e). This suggested that KLK4 overexpression induces molecular shifts toward an epithelial phenotype by regulating two core EMT markers (E‑cadherin and N‑cadherin). It should be noted that our analysis only detects two core EMT markers, and altered expression of these proteins cannot fully prove complete suppression of the entire EMT program. These findings indicate that KLK4 overexpression significantly inhibits LSCC cell proliferation, promotes apoptosis, and alters expression of EMT markers, suggesting that KLK4 plays a crucial role in regulating tumor cell behavior.

### Regulation of Wnt/β-Catenin Signaling Pathway by KLK4 in LSCC Cells

**Fig. 4:**
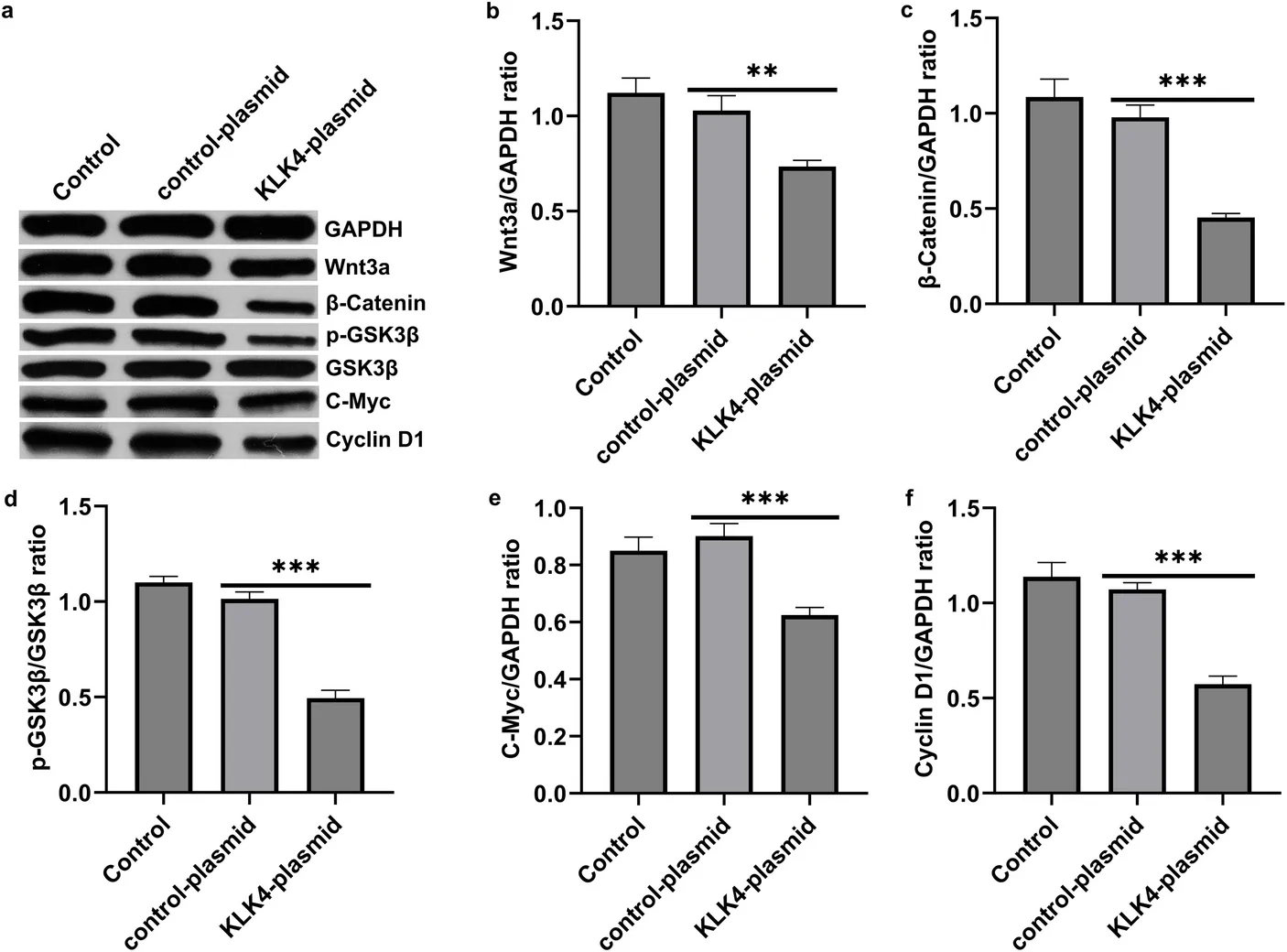
Regulation of the Wnt/β-catenin signaling pathway by KLK4 in LSCC cells. **a** Western blot analysis of Wnt3a, β-Catenin, p-GSK3β, GSK3β, C-Myc, and Cyclin D1 protein levels in AMC-HN-8 cells transfected with control-plasmid or KLK4-plasmid. **b** Wnt3a/GAPDH ratio. **c** β-Catenin/GAPDH ratio. **d** p-GSK3β/GSK3β ratio. **e** C-Myc/GAPDH ratio. **f** Cyclin D1/GAPDH ratio. Data are from three independent experiments (*n* = 3). **p < 0.01; ***p < 0.001

The Wnt/β-catenin signaling pathway plays a crucial role in cell proliferation, differentiation, and survival, and its abnormal activation is associated with the development and progression of various cancers. Prior mechanistic studies in gastric cancer and oral carcinomas have confirmed functional crosstalk between KLK4 and the Wnt/β-catenin cascade, where KLK4 modulates pathway activity to drive malignant phenotypes in those tumor models. However, the precise role of KLK4 in LSCC remains unclear, particularly whether it influences tumor cell behavior by regulating the Wnt/β-catenin pathway. To further investigate the regulatory effect of KLK4 on the Wnt/β-catenin signaling pathway in LSCC cells, we transfected AMC-HN-8 cells with either the control or KLK4 plasmid. The expression levels of key components in the Wnt/β-catenin pathway, including Wnt3a, β-catenin, GSK3β, p-GSK3β, C-Myc, and Cyclin D1, were assessed after 24 h of transfection using western blotting. The results showed that, compared to the control-plasmid group, overexpression of KLK4 significantly reduced the levels of Wnt3a, β-catenin, and p-GSK3β, while also suppressing downstream targets C-Myc and Cyclin D1. However, the total GSK3β levels remained unchanged (Fig. 4a–f). KLK4 overexpression significantly reduced Wnt3a expression and decreased the phosphorylation level of GSK3β (p-GSK3β) without affecting total GSK3β. The reduced p-GSK3β enhanced GSK3β activity, which accelerated the degradation of β-catenin, leading to prominently decreased β-catenin protein levels. Downstream targets including C-Myc and Cyclin D1 were also inhibited. These data suggest that KLK4 may suppress LSCC cell proliferation in association with the reduced activity of the Wnt/β-catenin signaling pathway.

### The Wnt/β-Catenin Pathway Is Involved in KLK4-Mediated Regulation of Proliferation, Apoptosis and EMT in LSCC Cells

**Fig. 5:**
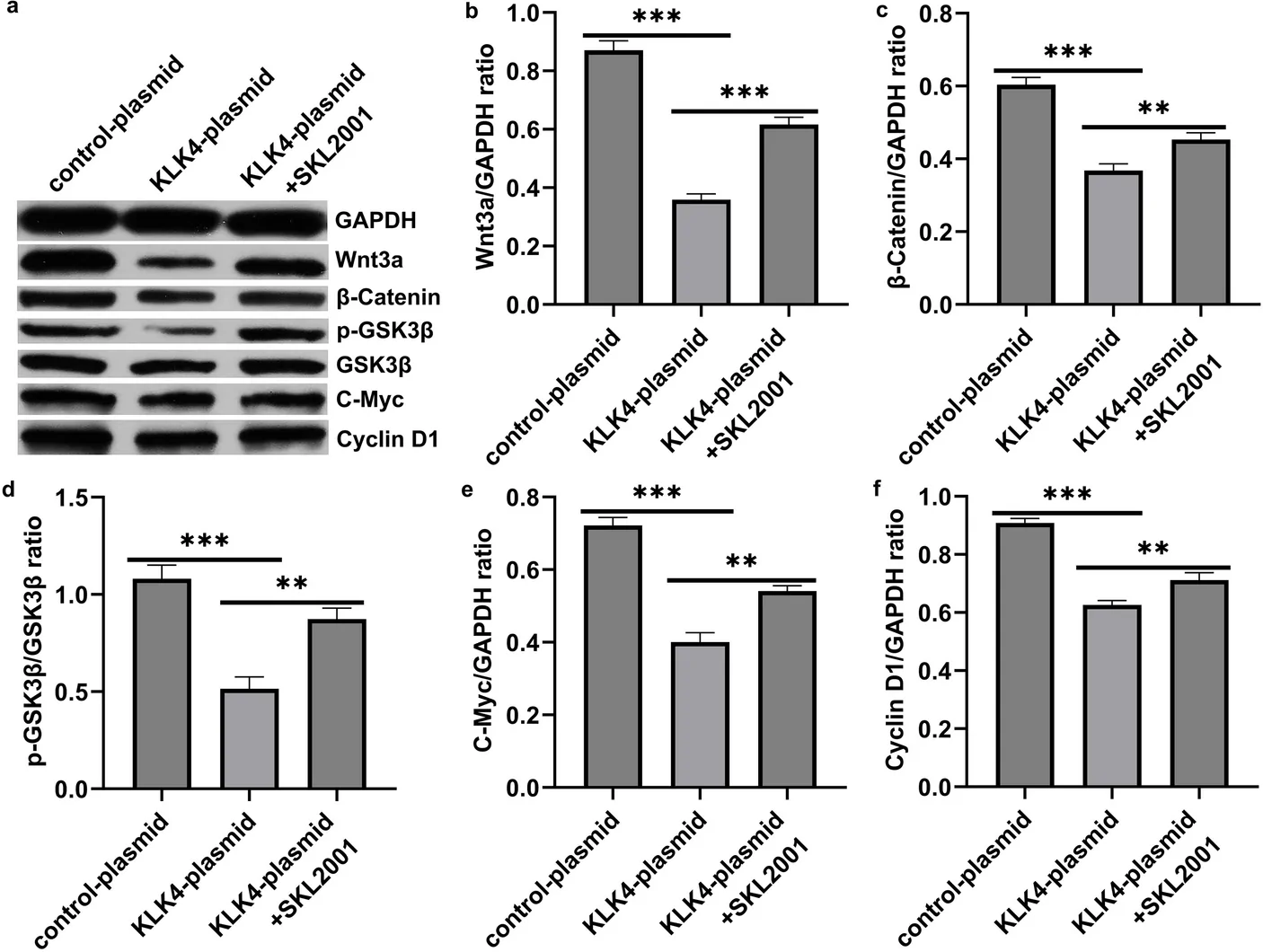
SKL2001 enhanced Wnt/β-catenin signaling in KLK4-plasmid transfected LSCC cells Western blot analysis of Wnt3a, β-Catenin, p-GSK3β, GSK3β, C-Myc, and Cyclin D1 protein levels in AMC-HN-8 cells transfected with KLK4-plasmid in the presence or absence of Wnt/β-catenin agonist SKL2001 (40 µM). Data are from three independent experiments (*n* = 3). **p < 0.01; ***p < 0.001

We transfected KLK4 plasmids into AMC-HN-8 cells and treated them with the Wnt/β-catenin pathway agonist SKL2001 (40 µM) after transfection for 24 h to investigate the role of the Wnt/β-catenin signaling pathway in KLK4-mediated regulation of LSCC cells. Western blot analysis (Fig. 5a–f) revealed that KLK4 overexpression significantly downregulated the protein levels of Wnt3a, β-catenin, p-GSK3β, C-Myc, and Cyclin D1 compared to the control-plasmid group. However, after SKL2001 treatment, the expression of these proteins was significantly increased, demonstrating that the Wnt/β-catenin pathway is pivotal in KLK4-mediated regulation.

**Fig. 6:**
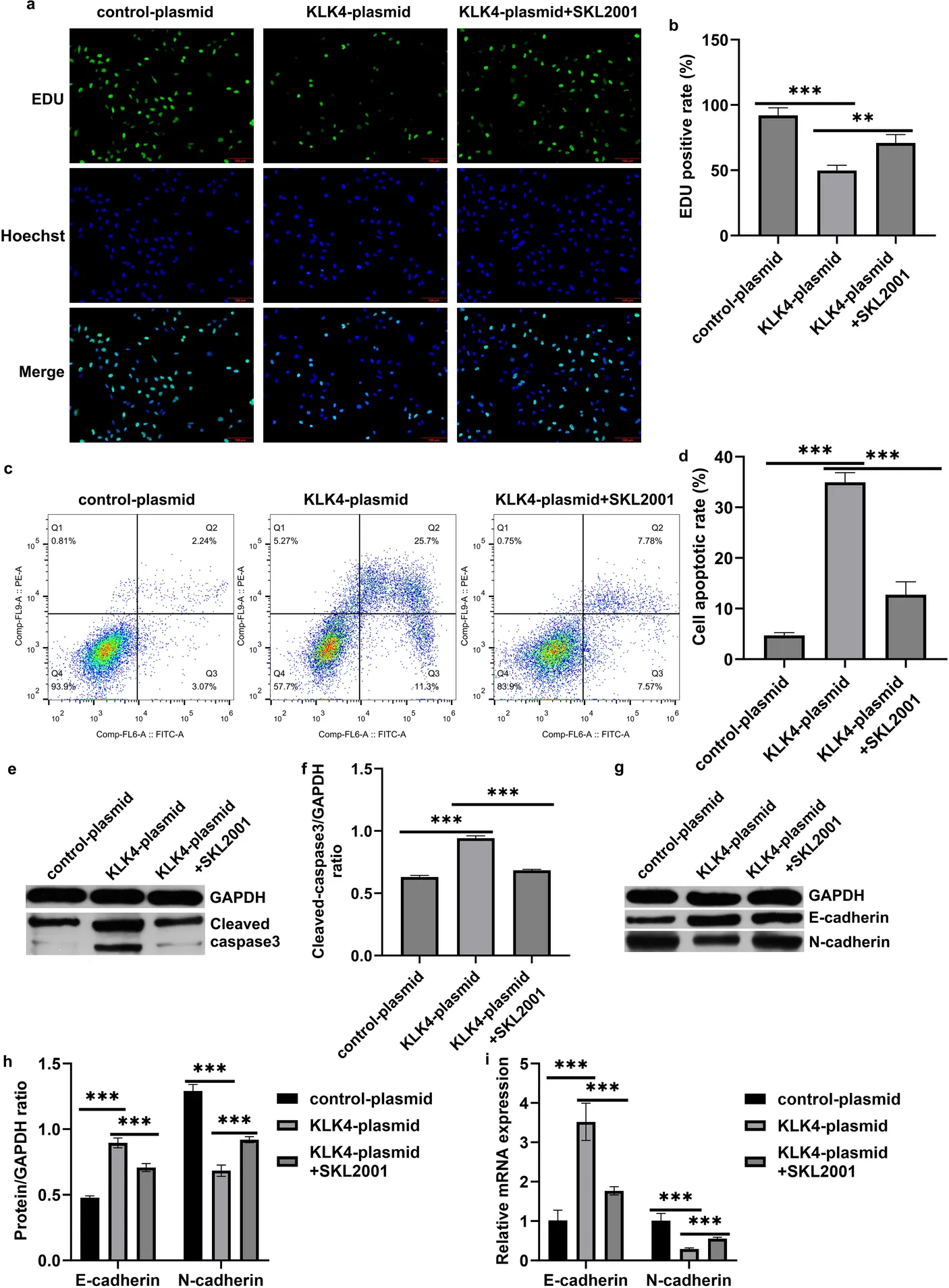
Effects of the Wnt/β-catenin pathway on KLK4-induced proliferation, apoptosis, and the expression of EMT-associated markers in LSCC cells. **a** and **b** EdU assay showing the cell viability in the different groups; **c** Flow cytometry analysis of apoptosis rates; **d** Quantification of total apoptotic cell percentage (sum of early apoptotic and late apoptotic populations) detected by Annexin V/PI flow cytometry; **e** and **f** Western blot analysis of cleaved-Caspase3 expression; **g**–**i** Western blot analysis and RT-qPCR of E-cadherin and N-cadherin expression. Data are from three independent experiments (*n* = 3). **p < 0.01; ***p < 0.001

EdU assay results showed that KLK4 overexpression significantly inhibited cell proliferation, whereas SKL2001 treatment restored the proliferative capacity (Fig. 6a and b). This suggested that KLK4 suppresses LSCC cell proliferation by inhibiting the Wnt/β-catenin signaling pathway, confirming the impact of KLK4 on LSCC cell proliferation. Furthermore, flow cytometry analysis demonstrated that KLK4 overexpression notably increased the apoptosis rate, an effect that was reversed by SKL2001, which significantly reduced apoptosis (Fig. 6c and d). Western blot analysis further confirmed the pro-apoptotic effect of KLK4, as shown by the increased expression of the cleaved-Caspase3 protein. SKL2001 treatment decreased cleaved-Caspase3 expression (Fig. 6e and f), reinforcing the anti-apoptotic role of the Wnt/β-catenin pathway.

Regarding EMT, RT-qPCR, and western blot analyses showed that KLK4 overexpression led to the upregulation of E-cadherin and downregulation of N-cadherin (Fig. 6g–i). The addition of SKL2001 effectively reversed these EMT marker changes, indicating that Wnt/β-catenin pathway activation reshapes epithelial-mesenchymal molecular profiles in KLK4-overexpressing cells.

In conclusion, KLK4 modulates LSCC cell proliferation, apoptosis, and the expression of EMT-associated molecular markers by regulating the Wnt/β-catenin signaling pathway. The ability of the Wnt/β-catenin agonist SKL2001 to reverse these effects underscores the crucial role of this pathway in KLK4-mediated LSCC progression.

## Discussion

This study highlights the crucial regulatory role of KLK4 in LSCC, particularly its influence on the Wnt/β-catenin signaling pathway. Our data suggest that KLK4 exerts tumor-suppressive effects in LSCC, including suppressed cell proliferation, enhanced apoptosis and inhibited EMT. These phenotypes are closely associated with reduced activity of the Wnt/β-catenin signaling pathway, indicating the antagonism of the Wnt/β-catenin signaling pathway represents one of the mechanisms underlying the protective effects of KLK4. Moreover, the Wnt/β-catenin pathway agonist SKL2001 partially reversed KLK4-mediated inhibition of cell proliferation and EMT, further underscoring the pivotal role of this pathway in KLK4-mediated regulation of LSCC.

Previous studies have shown that aberrant activation of the Wnt/β-catenin pathway is closely associated with increased cell proliferation, enhanced invasiveness, and poor prognosis in patients with LSCC (Shiah et al. 2015; Zhang and Wang 2020). Several components of this pathway have been identified as potential therapeutic targets. Our study supports this therapeutic strategy by demonstrating that targeting the Wnt/β-catenin pathway can modulate the effects of KLK4 on LSCC cell behavior. Specifically, our results indicate that KLK4 expression is correlated with altered activity of the Wnt/β-catenin pathway, accompanied by downregulation of Wnt3a, decreased GSK3β phosphorylation and reduced β-catenin levels. These alterations jointly suppress pathway activation, whose downstream transcription targets mediate cell proliferation and survival (MacDonald et al. 2009). This suggested that KLK4 exerts tumor-suppressive effects by negatively regulating oncogenic pathways.

As a secreted serine protease, KLK4 may suppress Wnt/β-catenin signaling via several literature-supported extracellular mechanisms worthy of future validation. Secreted KLK4 could proteolytically degrade extracellular Wnt3a ligand to reduce ligand bioavailability for membrane receptors, analogous to other secreted proteases that inactivate Wnt ligands in the tumor microenvironment (He and Tang 2020). KLK family proteases also remodel ECM proteoglycans to accelerate Wnt ligand clearance within the tumor microenvironment (Kontos and Scorilas 2012; Borgoño and Diamandis 2004). Alternatively, KLK4 may modulate PAR1-mediated paracrine cytokine signaling to indirectly restrain canonical Wnt activity (Wang et al. 2010). Our observation of reduced Wnt3a mRNA upon KLK4 overexpression also suggests potential intracellular transcriptional repression of Wnt3a, combining with extracellular proteolytic effects to jointly suppress pathway activation.

Furthermore, our study revealed that KLK4 promotes apoptosis in LSCC cells. The effect of KLK4 on apoptosis was diminished by SKL2001 treatment, suggesting that the Wnt/β-catenin pathway may have an anti-apoptotic role in this context. This observation is consistent with previous reports of the anti-apoptotic effects of the Wnt/β-catenin signaling pathway in various cancers (Zhu et al. 2023). Therefore, the regulation of apoptosis by KLK4 in LSCC may involve complex interactions with multiple signaling pathways, warranting further investigation.

EMT is the process of transforming epithelial cells into mesenchymal phenotypic cells, which is mainly manifested by the decreased expression of E-cadherin in epithelial cells and increased expression of N-cadherin in interstitial cells, thereby changing the adhesion properties of cells and promoting cell migration and invasion (Lamouille et al. 2014). Our study revealed that KLK4 overexpression regulates the expression of canonical EMT molecular markers to shift cells toward an epithelial molecular phenotype, as evidenced by increased E-cadherin and decreased N-cadherin. EMT is a critical process in cancer metastasis, and its inhibition is typically associated with reduced metastatic potential (Zhang and Weinberg 2018). The ability of SKL2001 to reverse the EMT-suppressive effects of KLK4 suggested that the Wnt/β-catenin pathway plays a key role in mediating EMT in LSCC. Previous studies have also linked the Wnt/β-catenin signaling pathway to the promotion of EMT and metastatic behavior in LSCC and other cancers (Carron et al. 2021; Meng et al. 2022).

As the Wnt agonist SKL2001 only partially rescued the malignant phenotypes suppressed by KLK4 overexpression, the tumor-regulatory functions of KLK4 in LSCC cannot be fully attributed to Wnt/β-catenin inhibition alone. This partial rescue indicates that KLK4 likely exerts anti-tumor effects via additional Wnt-independent signaling cascades in laryngeal cancer cells. Future multi-omics screening will help identify other downstream molecular pathways mediated by KLK4 to fully dissect its comprehensive regulatory network.

However, this study had several limitations. First, all functional analyses were conducted using KLK4 overexpression alone, with no complementary KLK4 knockdown assays. This absence of loss-of-function data limits our ability to fully confirm the role of endogenous KLK4 in LSCC. Second, all core functional validation experiments were solely carried out in AMC-HN-8 cells, without phenotype verification in the other two LSCC cell lines tested in this study. In addition, we lacked in vivo animal models to further confirm our in vitro observations. The absence of multi-cell-line validation and in vivo evidence may limit the generalizability of our conclusions. Third, the current evidence based on expression analysis and SKL2001 pharmacological intervention only reveals a correlative relationship between KLK4 and the Wnt/β-catenin pathway, rather than confirming direct molecular interaction between KLK4 and pathway components. The precise molecular mechanism underlying this association remains unclear. Fourth, we confirmed decreased KLK4 mRNA and protein abundance in LSCC serum and tissue specimens from a cohort of only 30 patients, yet we did not conduct diagnostic performance assessment or correlation analysis between KLK4 expression and clinical pathological parameters including tumor stage, differentiation grade and lymph node metastasis. Given the limited sample size and absence of supporting clinical statistical data, KLK4 cannot be defined as a validated diagnostic or prognostic biomarker for LSCC progression at this stage. Our expression results only deliver preliminary hints for subsequent large independent cohort studies to comprehensively verify the clinical biomarker and therapeutic potential of KLK4. Besides, vehicle control and SKL2001-only group were not set up in the rescue experiments.

To address these shortcomings in future research, we plan to perform KLK4 knockdown experiments and repeat key functional assays in multiple LSCC cell lines. We will also explore the molecular interaction between KLK4 and the Wnt/β-catenin pathway, conduct in vivo validation and clinical correlation analysis to enrich and improve the present conclusions. Further high-throughput analyses such as single-cell RNA sequencing (scRNA-seq) will be applied in our follow-up work to systematically screen other potential downstream pathways regulated by KLK4 in LSCC.

In summary, our study demonstrates that KLK4 exerts tumor-suppressive effects in LSCC, and these biological functions are closely associated with the Wnt/β-catenin signaling pathway, highlighting its potential as a novel biomarker and therapeutic target.

## Conclusion

The Wnt/β-catenin signaling pathway is involved in KLK4-mediated modulation of proliferation, apoptosis, and EMT in LSCC cells. Our data demonstrate a close association between KLK4 function and Wnt/β-catenin pathway activity in LSCC. These preliminary results indicate that KLK4 and the Wnt/β-catenin pathway could act as candidate targets worthy of further clinical and pharmacological verification for LSCC treatment.

## Supplementary Information

Below is the link to the electronic supplementary material.

Supplementary Material 1

## Data Availability

The datasets used and/or analyzed during the current study are available from the corresponding author on reasonable request.
