# Supplementary material for: Kallikrein-Related Peptidase 4 Inhibits Laryngeal Squamous Cell Carcinoma Potentially via Involvement of the Wnt/β-Catenin Signaling Pathway
Source: Biochem Genet. 2026 Aug 20;64(6):8492–508. doi: 10.1007/s10528-026-11443-w (PMC13723506; doi:10.1007/s10528-026-11443-w)
Supplement: Supplementary file 1 [file 10528_2026_11443_MOESM1_ESM.docx]

**Original blots for Figure 1d**

**Original blots for Figure 2b**

**Original blots for Figure 2h**

**Original blots for Figure 3a**

**Original blots for Figure 4a**

**Original blots for Figure 5a**

**Original blots for Figure 6e**

**Original blots for Figure 6g**
